# Dissemination of knowledge from Cochrane Public Health reviews: a bibliographic study

**DOI:** 10.1186/s13643-023-02272-8

**Published:** 2023-07-03

**Authors:** Stefanie Maria Helmer, Katja Matthias, Lea Mergenthal, Mia Reimer, Karina Karolina De Santis

**Affiliations:** 1 Institute of Public Health and Nursing Research, Faculty 11 Human and Health Sciences, University of Bremen, Grazer Str. 4, 28359 Bremen, Germany; 2 Cochrane Public Health Europe (https://ph.cochrane.org/cochrane-public-health-europe), Bremen, Germany; 3 Faculty of Electrical Engineering and Computer Science, University of Applied Science Stralsund, Stralsund, Germany; 4 Department of Prevention and Evaluation, Leibniz Institute for Prevention Research and Epidemiology—BIPS, Bremen, Germany

**Keywords:** Dissemination, Knowledge translation, Stakeholder, Systematic review, Cochrane, Public health, Meta-research, Bibliographic study

## Abstract

**Background:**

Appropriate dissemination of public health evidence is of high importance to ensure that scientific knowledge reaches potential stakeholders and relevant population groups. A wide distrust towards science and its findings indicates that communication thereof remains below its potential. Cochrane Public Health provides an important source of high-quality scientific evidence in the field of public health via reviews with systematic methodology. The aims of this study were to identify (1) dissemination strategies and (2) stakeholders of Cochrane Public Health reviews.

**Methods:**

This is a bibliographic study with a cross-sectional design. All 68 records (reviews or review protocols) listed on the Cochrane Public Health website (https://ph.cochrane.org/cph-reviews-and-topics) up to 8 March 2022 were included. Record characteristics, dissemination strategies, and potential stakeholder details were coded by one author, and 10% of records were checked by another author. Data were analyzed using descriptive statistics or narratively into common themes.

**Results:**

The 68 records were published between 2010 and 2022 and included 15 review protocols and 53 reviews with systematic methodology (46 systematic, 6 rapid, and 1 scoping review). All 53 reviews were disseminated via open-access plain language summaries (PLS) in English with translations into 3–13 other languages. Other dissemination strategies included information on Cochrane websites (e.g., clinical answers or guidelines) available for 41/53 reviews and Cochrane news or blogs that mentioned 19/53 reviews. Overall, 23/68 records mentioned the actual stakeholder involvement in review production, protocol development, or formulation of dissemination plans. The potential stakeholders included several highly diverse groups, such as the general population or specific communities (e.g., racial minority groups), policy and decision makers, and researchers and professionals in various fields (e.g., nutrition, physical activity, education, or care).

**Conclusions:**

This study shows that Cochrane Public Health reviews are disseminated predominantly via PLS in different languages and via review information on Cochrane websites. Planned dissemination strategies were rarely reported although actual stakeholders were involved in the planning and production of some reviews. The relevance of Cochrane Public Health reviews for non-academic stakeholders and the general population highlights the need for the dissemination of evidence from such reviews beyond academia.

**Systematic review registration:**

The study was prospectively registered at the Open Science Framework (https://osf.io/ga9pt/).

**Supplementary Information:**

The online version contains supplementary material available at 10.1186/s13643-023-02272-8.

## Background

“Scientific papers sit in archives, Matters of indifference to almost everyone”

Richard Powers, “The Overstory,” 2019, USA: Norton & Company

A research-to-practice gap addressed in the quote above means that scientific results often do not reach the potential stakeholders and the general population [1, 2]. According to the World Health Organization, bridging the so-called “know-do gap” or “research-to-practice gap” is one of the most important challenges for research in medicine and public health [3].

To reduce such a gap and to achieve an improvement in science communication, dissemination as well as knowledge translation are two essential concepts that need to be considered. Dissemination is defined as “an active approach of spreading evidence-based interventions to the target audience via predetermined channels using planned strategies” ([4], p. 118) and is the foundation for the implementation of interventions by mostly non-research audiences [5]. Effective strategies to facilitate dissemination include establishing collaborations and personal relationships with practitioners and policy makers [6], educational outreach visits [7] or dissemination programs and strategies that are tailored to determinants of practice [8]. Knowledge translation is described as “a dynamic and iterative process that includes the synthesis, dissemination, exchange, and ethically sound application of knowledge to improve health, provide more effective health services and products, and strengthen the health care system” ([9], p. 165). For the purposes of this study, we refer to dissemination as a broad umbrella term consisting of both terms (dissemination and knowledge translation).

Research shows that professionals in practice know about the importance of dissemination, however, the processes of dissemination do not seem to function effectively [10]. For example, although practitioners know and use scientific databases to obtain scientific information regarding their field of interest, discrepancies between information needs and information use have been reported [11]. Concepts that could improve dissemination are that communication should be conducted in a simple way and that various aspects of such communication (i.e., the message content, its communicator, and the method of message translation) need to be acknowledged on a local and community level [12]. Therefore, the message, the source, the audience, and the channel need to be carefully tailored towards the needs of the stakeholders [10]. Moreover, there is a need for an infrastructure to facilitate the distribution of scientific research by multiple internet-based communication channels, including online videos and virtual exchanges, podcasts, blogs, and tweets [13].

The COVID-19 pandemic highlighted the need for an effective dissemination of knowledge from public health research directly to any stakeholders, including academic and non-academic audiences and the general population. The Cochrane Collaboration, that is an important source of high-quality health-related syntheses of scientific findings, recognized that scientists have struggled to properly communicate their research beyond the scientific community [12] and that this lack of adequate scientific communication contributed to a wide distrust towards science and its findings. Hence, the dissemination of knowledge was listed as one of the research priorities for Cochrane in 2022 [14]. Among others, Cochrane aims in “investing in science communications which will strengthen our ability to communicate uncertainty in a way citizens understand, as well as being more proactive about science communication” [14].

The broad objective of this study is to assess how authors of reviews with systematic methodology in public health address dissemination in their reviews. The source of reviews for this study is a relevant division of Cochrane (i.e., Cochrane Public Health [15]). We selected this source because Cochrane Public Health reviews cover a broad range of topics and thus are likely to be relevant for heterogeneous stakeholder groups. However, it is unclear if and how authors plan and address dissemination during the review production. Therefore, the aims of this study were to identify (1) dissemination strategies and (2) stakeholders of Cochrane Public Health reviews.

## Methods

### Study design and setting

This is a bibliographic study with a cross-sectional design. A protocol for this study was prospectively registered at the Open Science Framework [16]. The study adheres to the Strengthening the Reporting of Observational Studies in Epidemiology (STROBE) guideline [17]. The STROBE checklist for this study is reported Additional file 1 (Table S1). This study was conducted at an academic public health setting in 2022. There were no changes between the protocol and the content of this study.

### Data source

The source of data for this study was the Cochrane Public Health website [15]. The inclusion criteria were as follows: (1) all records (i.e., review protocols or reviews with systematic methodology: systematic, rapid, or scoping) available on the Cochrane Public Health website [15] up to 8 March 2022 and (2) the newest records in case reviews with existing protocols or review updates were published. The exclusion criteria were as follows: (1) the original review in case review update was published and (2) the review protocol in case review with review protocol was published. The exclusion criteria were chosen to reduce bias in this study by preventing duplication of records.

### Data collection process

All records on the Cochrane Public Health website [15] were identified by one author, imported into EndNoteX9 (Clarivate) for study management, downloaded as full text documents, and stored for further processing in a pdf format. A data spreadsheet (Excel 10, Microsoft Inc.) and a data coding manual (Additional file 1: Table S2) were developed by one author and calibrated in the team. One author coded all data from all records. To reduce bias in data coding, another author checked the coding in 10% of records. Any discrepancies were discussed between the two authors, and a consensus was reached by discussion. Since no major discrepancies between both authors were detected, no further data checks were performed.

### Variables (data items) and measurement

A list of variables (data items) necessary to address the aims of this study was developed by all authors (Table 1).

**Table 1:** Data items coded in this study

Data item	Item content	Coded information
(1)	Bibliographic information	First author, publication year, author region, title, aim
(2)	Record characteristics	Type (review or review protocol), review type (systematic, rapid or scoping), number of primary studies included in the review
(3)	Research question details	Description of population, intervention focus, and intervention setting based on title and aim
(4)	Dissemination plan	Existence of a dissemination plan with objectives, audience, or timeline included in the record (yes/no)
(5)	Dissemination via plain language summary (PLS)	PLS included in the record (yes/no), open-access PLS (yes/no), PLS translation from English to other languages (yes/no; if yes, list and number of languages)
(6)	Dissemination via other strategies	Description of other strategies mentioned in reviews or planned in review protocols
(7)	Stakeholder involvement	Advisory group involvement in protocol development (yes/no) or in review production (yes/no)
(8)	Potential stakeholders	Identified based on population, intervention focus, and intervention setting in abstracts

The data were coded either quantitatively into predefined categories (e.g., yes or no) or qualitatively using information from the records (e.g., study aims from the abstract). To identify dissemination strategies, we searched any Cochrane websites for information on each included record and full texts of all included records. We read the abstracts, methods, conclusion, and acknowledgement sections of each record and screened them for the following keywords: “disseminat*,” “knowledge trans*,” “patient involve*,” “communicat*,” “advisory,” “public representative,” “patient representative,” “community representative,” “stakeholder,” “policy,” and “consumer.” We also screened for these keywords in the full text of each record using the search function in Adobe Reader.

### Data synthesis

The data file is reported in Additional file 2. The coded data were synthesized using descriptive statistics (e.g., relative frequencies in Excel 10) or narratively into common themes that inductively emerged from the data. For example, the information from record titles and aims was read by one author and clustered into common themes to identify the population (e.g., healthy adults from the general population), intervention focus (e.g., physical activity promotion), and intervention setting (e.g., any setting applicable to the general population). Stakeholders were defined as (1) actual stakeholders (e.g., advisory groups) who, in addition to review authors, were involved in the review (e.g., provided feedback on review protocol) and (2) potential stakeholders for whom the review results could be relevant (e.g., nutrition specialists). The actual stakeholders were identified based on information about any advisory group mentioned in record full texts, and potential stakeholders were identified based on population, intervention focus, and intervention setting in each record.

### Dissemination of this study

We planned to disseminate the results of this study using the following strategies: (1) academic publications (a conference abstract and this article), (2) plain language summaries in English and in German, and (3) online presentations for Cochrane Public Health editors.

## Results

### Sample size

This study includes data from all 68 records listed in Additional file 1 (Table S3) that were available on the Cochrane Public Health website [15] up to 8 March 2022. No records were excluded. There was no duplication among the records because review updates were listed only once (i.e., the original reviews were not available on the website) and review protocols were listed only for reviews in progress (i.e., review protocols for published reviews were not available on the website).

### Characteristics of the included records (review protocols and reviews)

The 68 records included 53 reviews with 1–153 primary studies and 15 review protocols (Table 2). The records were published between 2010 and 2022, and 54.4% originated from Europe (Table 2). Among the 53 reviews, 86.8% were systematic reviews and 48.5% conducted a meta-analysis (Table 2).

**Table 2:** Characteristics of the included records (*n* = 68)

Variables	Records (*n* = 68)	
	***n***	**%**
Record type		% of 68
Review	53	77.9
Review protocol	15	22.1
Region of the corresponding author		% of 68
Europe	37	54.4
Australia	13	19.1
North America	12	17.6
Africa	3	4.4
Asia	3	4.4
Meta-analysis conducted		% of 68
Yes	33	48.5
No	19	27.9
Not applicable (review protocol or scoping review)	16	23.5
Review type		% of 53
Systematic	46	86.8
Rapid	6	11.3
Scoping	1	1.9

The research questions were based on the Population, Intervention, Comparison, and Outcome (PICO) framework in 65/68 (95.6%) records and on the Population, Concept, and Context (PCC) framework in 3/68 (4.4%) records. The 68 records included predominantly populations of any age (63.2%) and any health status (94.1%; Table 3). The interventions in 68 records focused predominantly on three themes: (1) food (e.g., fortification in the context of micronutrient deficiency), (2) healthy lifestyle (e.g., physical activity promotion), and (3) social participation (e.g., protection of social minorities; Table 3). The settings of these interventions included predominantly any settings applicable to the general population or to specific communities (e.g., racial minorities; Table 3).

**Table 3:** Population, intervention, and setting in the included records (*n* = 68)

Variables	Records (*n* = 68)	
	*n*	% of 68
Population by age		
Any (adults and children)	43	63.2
Only adults	14	20.6
Only children	11	16.2
Population by health status		
Any (healthy and clinical)	64	94.1
Only healthy	3	4.4
Only clinical	1	1.5
Intervention focus		
Food (fortification, labeling, taxation, marketing)	22	32.4
Healthy lifestyle (physical activity, weight control, diet, tobacco, and alcohol use control)	13	19.1
Social participation (inclusion, protection, equality, financial incentives)	12	17.6
Policy and environment (policy development, implementation, decision-making, environmental pollution)	8	11.8
Any health focus or specific health focus (drowning, household risks, housing, oral health)	7	10.3
COVID-19	6	8.8
Intervention setting		
Any settings applicable to the general population	33	48.5
Any settings applicable to specific communities (racial, ethnic, and social minorities, low- to middle-income countries, high-income countries, elderly, obese, households, housing)	18	26.5
School and childcare	9	13.2
Digital and other media-based settings	4	5.9
Other specific settings (workplace, care facility, sports organization)	4	5.9

### Dissemination strategies

A dissemination plan (i.e., a list of planned activities designed to disseminate the findings of the review) was included in 3/68 (4.4%) records [18–20] (Table 4). The dissemination plans included information about informing experts in the field to ensure “that evidence-based recommendations were disseminated widely, and, where possible, implemented” ([18], p. 17), about “the opportunities for dissemination to a range of non-academic audiences” ([19], p. 135) or involved expert groups in the review production (e.g., policy advisers and civil society representatives) who provided feedback “that the review will appropriately inform policy” ([20], p. 293).

**Table 4:** Dissemination strategies in the included records (*n* = 68; *n*_review_ = 53, *n*_review protocol_ = 15))

Variables	Records (*n* = 68)	
	*n*	%
Dissemination plan included in the record		% of 68
Yes	3	4.4
No	65	95.6
Dissemination via plain language summary (PLS)		
Yes (review)	53	100.0
Yes (review protocol)	0	0.0
Plain language summary (PLS)		
Open-access	53	100.0
Translated from English to at least one other language (total of 3–13 languages)	53	100.0
Translation language		
German	53	100.0
Spanish	53	100.0
Dissemination via other strategies: review information on Cochrane Public Health or other Cochrane websites		% of 53 / % of 15
Yes (review)	41	77.4
Yes (review protocol)	0	0.0
Review information on Cochrane Public Health or other Cochrane websites		% of 41
Cochrane clinical answer	28	68.3
Special collection	15	36.6
Guideline	14	34.1
Podcast	13	31.7
Editorial	2	4.9
Dissemination via other strategies: cochrane news or blogs		% of 53 / % of 15
Yes (review)	19	35.8
Yes (review protocol)	0	0.0

Plain language summary (PLS) was used as a dissemination strategy for all reviews, while all review protocols did not have PLS (Table 4). All 53 reviews had open-access PLS in English with translations into 3–13 other languages. Overall, the PLS for all 53 reviews were available in English, German, and Spanish.

Other dissemination strategies included review information on Cochrane Public Health or Cochrane websites that were available for 77.4% of reviews in the format of clinical answers, special collections, guidelines, podcasts, or editorials (Table 4). Furthermore, 35.8% of reviews were disseminated via Cochrane news or blogs (Table 4).

### Stakeholders

The actual stakeholders were mentioned in 23/68 (33.8%) records. These records mentioned that advisory groups were involved (1) in review production (in 19/23 records), (2) in protocol development (in 12/23 records), or (3) in formulation of dissemination plans (in 3/23 records included in Table 4).

The potential stakeholders included several highly diverse groups for whom review results could be relevant based on the population, intervention focus, and intervention setting (all reported in Table 3). The potential stakeholders included the general population, population in specific communities (e.g., racial minority groups), policy and decision makers, and researchers and professionals in various fields (e.g., nutrition, physical activity, education, care, infection protection, community management, work, sport, media and technology, or environment protection).

### Dissemination of this study

As planned, we disseminated the results of this study using the following strategies: (1) academic publications (a conference abstract [21] and this article), (2) PLS in English and in German (Additional file 1: Table S4), and (3) informal online presentations for Cochrane Public Health editors and for a virtual satellite group of Cochrane Public Health in German-speaking countries in Europe (Cochrane Public Health Europe [22]).

## Discussion

This bibliographic study based on 68 records (review protocols or reviews) shows that Cochrane Public Health reviews are predominantly disseminated via plain language summaries (PLS) available open-access and in multiple languages on the Cochrane Public Health website [15]. Other dissemination strategies included review information on Cochrane websites and Cochrane news or blogs. Planned dissemination strategies were rarely reported although actual stakeholders were involved in planning and production of some reviews. Cochrane Public Health records focused on various themes, such as food, healthy living, and social participation. Thus, these records could be of interest to several highly heterogenous stakeholder groups, including the general population and non-academic audiences.

Systematic reviews provide the best available evidence that should serve as the basis for evidence-based practice. Dissemination strategies should be chosen to enable consumers as well as practitioners to identify key messages by using the appropriate language and the appropriate product [2, 23]. Hence, the inclusion and translation of PLS in well-conducted systematic reviews appear to be one of the most important dissemination strategies and a central task in the future to allow any stakeholders to find what they need regardless of their cultural background, their spoken language, or limited access to information [12]. In the last years, PLS were more commonly used in biomedical journals but PLS from the Cochrane Collaboration remained the most well-studied [24]. Our study shows that all Cochrane Public Health reviews included PLS in three languages (English, German, and Spanish) but also other languages were available. A randomized controlled trial showed that reading a PLS helped patients to understand the benefits and harms as well as the quality of evidence [25]. Moreover, evidence from systematic reviews highlighted that accessibility as well as early dissemination to stakeholders can maximize interest and applicability [26]. Furthermore, PLS can reduce identified knowledge translation barriers, such as reducing the time to read evidence sources [2]. Although all Cochrane Public Health reviews had a PLS in a format of a non-scientific text, other formats of PLS, such as infographics also exist. Infographics are user-friendly because they rely on the graphical representation of results rather than on the reading experience alone [27]. However, infographics may be more difficult and time-consuming to design than writing a text-based PLS for review authors.

The Cochrane Public Health reviews (i.e., review abstract, its PLS in English, and any PLS translations) are freely available on the Cochrane Public Health website [15]. The Cochrane Public Health reviews were also disseminated via different communication channels, including special collections, blogs, and podcasts that could be of relevance to the general population and non-academic stakeholders. Using a variety of channels seems appropriate as long as those match the targeted audiences [10]. It should be noted, that our analysis only included distribution channels that were mentioned on the Cochrane Public Health website [15]. Other channels that are of particular interest to practitioners and policy makers, such as news media, social media, or policy briefs [10] are also targeted by Cochrane via its social media presence. Still, most of the identified dissemination channels used a passive way of knowledge translation for which the recipients need to actively search for the information. Active knowledge translation by including stakeholders in the review production is less often used by academics and researchers [10], although advisory group involvement was mentioned in 23/68 (33.8%) records in this study. Moving from passive to active strategies could be one key priority in the future of science communication since research shows that active strategies, such as collaborations with practitioners [6] or educational outreach visits [7], are effective dissemination strategies. Research also shows that active knowledge translation in workshops or webinars can reduce the volume of research evidence for practitioners and can prevent misunderstandings of research evidence [2]. Stakeholder engagement in research and evidence synthesis processes is likely to enhance dissemination [28] and can support dissemination and active knowledge translation. Stakeholders can be engaged in various stages of review production, for instance in topic consultation, input meetings, and dissemination but also as review team members [28]. Stakeholders can furthermore contribute information relevant at a local level which is valued by public health professionals [29, 30]. Although some Cochrane Public Health reviews mentioned advisory boards, the details of their involvement in protocol development or review production were not explained. Only 3/68 (4.4%) records [18–20] included a dissemination plan aiming to inform non-academic audiences or policy. The reason for this result may be that the inclusion of dissemination plans is not mandatory in Cochrane Public Health reviews. A formal requirement for inclusion of dissemination plans in any Cochrane or non-Cochrane review could encourage authors to consider dissemination and the outreach of their work beyond the scientific community. However, inclusion of mandatory dissemination plans in reviews will not guarantee that such plans will be implemented post-publication. Review authors may also not know any relevant non-academic or policy platforms during review production and writing. Instead, in addition to using the predetermined and planned channels, as mentioned in the dissemination definition [4], dissemination could also be done post-publication. In fact, Cochrane offers various dissemination strategies and encourages authors to voluntarily disseminate their reviews post-publication, although it is unclear if review authors take up any such offers. In general, dissemination can be difficult because it requires additional time and resources and most academic researchers do not have a formal training in science communication [31]. Further research is required to identify other dissemination strategies for Cochrane reviews beyond PLS and the Cochrane website. Some support for review authors in the planning of stakeholder involvement in the review could be provided by theoretical frameworks [32]. Identification of best-practice examples of dissemination strategies or support tools, such as frameworks, could encourage review authors to use such strategies, even if resources are scarce.

### Limitations

There were several limitations in this study. First, we included records published by one institution (Cochrane) and in one field (public health) meaning that the results of this study may not be generalizable to other review publishers and to other academic fields. Second, we searched the reviews for specific keywords to find relevant information on dissemination and identify the relevant stakeholders. It cannot be ruled out that dissemination or stakeholders were addressed using other terms not included among our keywords, such as “end users.” Third, we did not systematically collect data on dissemination strategies post-publication beyond the Cochrane website. Thus, it is not clear if and how the evidence from Cochrane Public Health reviews was disseminated post-publication and who used such evidence and for what purposes. Fourth, data coding was done by one author and checked by another author in only 10% of records. Thus, it cannot be ruled out that some information was not coded or incorrectly coded, although no major discrepancies between both authors were detected in the checked 10% of records.

## Conclusions

This study shows that Cochrane Public Health reviews are disseminated predominantly via PLS in different languages and via review information on Cochrane websites. Planned dissemination strategies were rarely reported although actual stakeholders were involved in the planning and production of some reviews. The relevance of Cochrane Public Health reviews for non-academic stakeholders and the general population highlights the need for the dissemination of evidence from such reviews beyond academia. Further research is required to identify other dissemination strategies for Cochrane reviews beyond PLS and the Cochrane website.

## Supplementary Information

**Additional file 1: Table S1.** STROBE Checklist. **Table S2.** Data coding manual. **Table S3.** A list of 68 included records.** Table S4.** Plain language summary.

**Additional file 2. **Data file.

## Data Availability

The dataset supporting the conclusions of this article is included within the article and its additional file.

## Abbreviations

PCC: Population, concept, context
PICO: Population, Intervention, Comparison, Outcome
PLS: Plain language summary
STROBE: Strengthening the Reporting of Observational Studies in Epidemiology
