# Supplementary material for: Dissemination of knowledge from Cochrane Public Health reviews: a bibliographic study
Source: Syst Rev. 2023 Jul 3;12:113. doi: 10.1186/s13643-023-02272-8 (PMC10318803; doi:10.1186/s13643-023-02272-8)
Supplement: Supplementary file 1 — Additional file 1: Table S1. STROBE Checklist. Table S2. Data coding manual. Table S3. A list of 68 included records. Table S4. Plain language summary. [file 13643_2023_2272_MOESM1_ESM.pdf]

Additional file 1

Dissemination of knowledge from Cochrane Public Health reviews: A bibliographic study

Helmer S, Matthias K, Mergenthal L, Reimer M, De Santis K. Dissemination of knowledge from Cochrane Public Health reviews: A bibliographic study. Systematic Reviews. 2023.

Content

Table S1. STROBE Checklist 2
Table S2. Data coding manual 5
Table S3. A list of 68 included records 7
Table S4. Plain language summary 13

Table S1. STROBE Checklist

"Yes" means that the item was addressed under the same subheading in the manuscript.

Item	Item No	Recommendation	Page No.
**Title and abstract**	1	(*a*) Indicate the study's design with a commonly used term in the title or the abstract	yes
		(*b*) Provide in the abstract an informative and balanced summary of what was done and what was found	yes
**Introduction**			
Background/rationale	2	Explain the scientific background and rationale for the investigation being reported	yes
Objectives	3	State specific objectives, including any prespecified hypotheses	yes
**Methods**			
Study design	4	Present key elements of study design early in the paper	yes
Setting	5	Describe the setting, locations, and relevant dates, including periods of recruitment, exposure, follow-up, and data collection	yes
Participants	6	(*a*) *Cross-sectional study*—Give the eligibility criteria, and the sources and methods of selection of participants	yes (Data source)
Variables	7	Clearly define all outcomes, exposures, predictors, potential confounders, and effect modifiers. Give diagnostic criteria, if applicable	Yes, Table 1
Data sources/ measurement	8*	For each variable of interest, give sources of data and details of methods of assessment (measurement). Describe comparability of assessment methods if there is more than one group	yes (Data collection process)
Bias	9	Describe any efforts to address potential sources of bias	yes (Data source and Data collection process)

Study size	10	Explain how the study size was arrived at	N/A
Quantitative variables	11	Explain how quantitative variables were handled in the analyses. If applicable, describe which groupings were chosen and why	Yes (Data synthesis)
Statistical methods	12	(*a*) Describe all statistical methods, including those used to control for confounding	Yes (Data synthesis)
		(*b*) Describe any methods used to examine subgroups and interactions	N/A
		(*c*) Explain how missing data were addressed	N/A
		(*d*) *Cross-sectional study*—If applicable, describe analytical methods taking account of sampling strategy	N/A
		(*e*) Describe any sensitivity analyses	N/A
**Results**			
Participants	13*	(a) Report numbers of individuals at each stage of study—eg numbers potentially eligible, examined for eligibility, confirmed eligible, included in the study, completing follow-up, and analysed	Yes (sample size)
		(b) Give reasons for non-participation at each stage	N/A
		(c) Consider use of a flow diagram	N/A
Descriptive data	14*	(a) Give characteristics of study participants (eg demographic, clinical, social) and information on exposures and potential confounders	Yes, Table 2
		(b) Indicate number of participants with missing data for each variable of interest	N/A
		(c) *Cohort study*—Summarise follow-up time (eg, average and total amount)	N/A
Outcome data	15*	*Cohort study*—Report numbers of outcome events or summary measures over time	N/A
		Case-control study—Report numbers in each exposure category, or summary measures of exposure	N/A
		Cross-sectional study—Report numbers of outcome events or summary measures	*Yes, Table 3-4*

Main results	16	(*a*) Give unadjusted estimates and, if applicable, confounder-adjusted estimates and their precision (eg, 95% confidence interval). Make clear which confounders were adjusted for and why they were included	N/A
		(*b*) Report category boundaries when continuous variables were categorized	N/A
		(*c*) If relevant, consider translating estimates of relative risk into absolute risk for a meaningful time period	N/A
Other analyses	17	Report other analyses done—eg analyses of subgroups and interactions, and sensitivity analyses	N/A
**Discussion**			
Key results	18	Summarise key results with reference to study objectives	yes
Limitations	19	Discuss limitations of the study, taking into account sources of potential bias or imprecision. Discuss both direction and magnitude of any potential bias	yes
Interpretation	20	Give a cautious overall interpretation of results considering objectives, limitations, multiplicity of analyses, results from similar studies, and other relevant evidence	yes
Generalisability	21	Discuss the generalisability (external validity) of the study results	Yes (Limitations)
**Other information**			
Funding	22	Give the source of funding and the role of the funders for the present study and, if applicable, for the original study on which the present article is based	yes

Source: von Elm E, Altman DG, Egger M, Pocock SJ, Gøtzsche PC, Vandenbroucke JP. Strengthening the Reporting of Observational Studies in Epidemiology (STROBE) Statement: Guidelines for reporting observational studies. *BMJ*. 2007; 335(7624): 806. doi:10.1136/bmj.39335.541782.AD

Table S2. Data coding manual

Bibliographic information

1. first author name
2. publication year
3. corresponding author region (Europe, North America, Asia, Australia, South America, Africa)
4. title
5. aim in abstract (according to study authors)

Record characteristics:

6. record type (protocol or review)
7. review type (systematic, rapid, scoping)
8. meta-analysis conducted (yes or no)
9. number of primary studies included in review

Research question details (based on PICO or PCC in abstract)

10. Population (e.g. general population, ethnic minority groups)
11. Population by age (any, children, adults)
12. Population by health status (any, healthy including at risk for disease, clinical with existing chronic illness)
13. Intervention description from title or aim
14. Intervention setting based on intervention description
15. Concept (if PICO not used or NA)
16. Context (if PICO not used or NA)

Dissemination strategies

17. PLS (plain language summary): included (yes, no)
18. PLS open-access (OA): yes, no
19. PLS translated: yes, no
20. PLS languages: list languages other than English
21. Dissemination via other strategies (record information on any Cochrane website), e.g. podcast
22. Dissemination strategies (other): mentioned in reviews or planned in protocols

a. Terms for search in full-text of records: keywords: "disseminat*", "knowledge trans*", "patient involve*", "communicat*", “advisory”, “public representative”, “patient representative”, “community representative”, ”stakeholder”, “policy”, and “consumer”.
b. NOTE: copy and paste author statement, include citation page (e.g., p. 10)

23. Stakeholder involvement in protocol development or review production
24. Potential stakeholders: identified based on PICO or PCC (in abstract)

Table S3. A list of 68 included records

Record type	Citation
**Systematic reviews**	[1], [2], [3], [4], [5], [6], [7], [8], [9], [10], [11], [12], [13], [14], [15], [16], [17], [18], [19], [20], [21], [22], [23], [24], [25], [26], [27], [28], [29], [30], [31], [32], [33], [34], [35], [36], [37], [38], [39], [40], [41], [42], [43], [44], [45]
Rapid reviews	[46], [47], [48], [49], [50], [51], [52]
Scoping reviews	[53]
Review protocols	[54], [55], [56], [57], [58], [59], [60], [61], [62], [63], [64], [65], [66], [67], [68]

1. Anderson LM, Adeney KL, Shinn C, Safranek S, Buckner-Brown J, Krause LK. Community coalition-driven interventions to reduce health disparities among racial and ethnic minority populations. Cochrane Database Syst Rev. 2015;(6):Cd009905. doi: 10.1002/14651858.CD009905.pub2.
2. Baker PR, Francis DP, Soares J, Weightman AL, Foster C. Community wide interventions for increasing physical activity. Cochrane Database Syst Rev. 2015;1:Cd008366. doi: 10.1002/14651858.CD008366.pub3.
3. Baker PRA, Francis DP, Hairi NN, Othman S, Choo WY. Interventions for preventing abuse in the elderly. The Cochrane database of systematic reviews. 2016;2016(8):CD010321-CD010321. doi: 10.1002/14651858.CD010321.pub2.
4. Brown T, Moore TH, Hooper L, Gao Y, Zayegh A, Ijaz S, Elwenspoek M, Foxen SC, Magee L, O'Malley C *et al*. Interventions for preventing obesity in children. Cochrane Database Syst Rev. 2019;7(7):Cd001871. doi: 10.1002/14651858.CD001871.pub4.
5. Burns J, Boogaard H, Polus S, Pfadenhauer LM, Rohwer AC, van Erp AM, Turley R, Rehfuess E. Interventions to reduce ambient particulate matter air pollution and their effect on health. Cochrane Database Syst Rev. 2019;5(5):Cd010919. doi: 10.1002/14651858.CD010919.pub2.
6. Centeno Tablante E, Pachón H, Guetterman HM, Finkelstein JL. Fortification of wheat and maize flour with folic acid for population health outcomes. Cochrane Database Syst Rev. 2019;7(7):Cd012150. doi: 10.1002/14651858.CD012150.pub2.
7. Chastin S, Gardiner PA, Harvey JA, Leask CF, Jerez-Roig J, Rosenberg D, Ashe MC, Helbostad JL, Skelton DA. Interventions for reducing sedentary behaviour in community-dwelling older adults. Cochrane Database Syst Rev. 2021;6(6):Cd012784. doi: 10.1002/14651858.CD012784.pub2.
8. Coren E, Hossain R, Pardo Pardo J, Bakker B. Interventions for promoting reintegration and reducing harmful behaviour and lifestyles in street-connected children and young people. Cochrane Database Syst Rev. 2016;2016(1):Cd009823. doi: 10.1002/14651858.CD009823.pub3.
9. Crockett RA, King SE, Marteau TM, Prevost AT, Bignardi G, Roberts NW, Stubbs B, Hollands GJ, Jebb SA. Nutritional labelling for healthier food or non-alcoholic drink purchasing and consumption. Cochrane Database Syst Rev. 2018;2(2):Cd009315. doi: 10.1002/14651858.CD009315.pub2.
10. Dangour AD, Watson L, Cumming O, Boisson S, Che Y, Velleman Y, Cavill S, Allen E, Uauy R. Interventions to improve water quality and supply, sanitation and hygiene practices, and their effects on the nutritional status of children. Cochrane Database Syst Rev. 2013;(8):Cd009382. doi: 10.1002/14651858.CD009382.pub2.

11. Das JK, Salam RA, Mahmood SB, Moin A, Kumar R, Mukhtar K, Lassi ZS, Bhutta ZA. Food fortification with multiple micronutrients: impact on health outcomes in general population. Cochrane Database Syst Rev. 2019;12(12):Cd011400. doi: 10.1002/14651858.CD011400.pub2.
12. De Buck E, Vanhove AC, O D, Veys K, Lang E, Vandekerckhove P. Day care as a strategy for drowning prevention in children under 6 years of age in low- and middle-income countries. Cochrane Database Syst Rev. 2021;4(4):Cd014955. doi: 10.1002/14651858.Cd014955.
13. Dobbins M, Husson H, DeCorby K, LaRocca RL. School-based physical activity programs for promoting physical activity and fitness in children and adolescents aged 6 to 18. Cochrane Database Syst Rev. 2013;2013(2):Cd007651. doi: 10.1002/14651858.CD007651.pub2.
14. Durao S, Visser ME, Ramokolo V, Oliveira JM, Schmidt BM, Balakrishna Y, Brand A, Kristjansson E, Schoonees A. Community-level interventions for improving access to food in low- and middle-income countries. Cochrane Database Syst Rev. 2020;7(7):Cd011504. doi: 10.1002/14651858.CD011504.pub2.
15. Field MS, Mithra P, Peña-Rosas JP. Wheat flour fortification with iron and other micronutrients for reducing anaemia and improving iron status in populations. Cochrane Database Syst Rev. 2021;1(1):Cd011302. doi: 10.1002/14651858.CD011302.pub3.
16. Garcia-Casal MN, Peña-Rosas JP, De-Regil LM, Gwirtz JA, Pasricha SR. Fortification of maize flour with iron for controlling anaemia and iron deficiency in populations. Cochrane Database Syst Rev. 2018;12(12):Cd010187. doi: 10.1002/14651858.CD010187.pub2.
17. Gibson M, Thomson H, Banas K, Lutje V, McKee MJ, Martin SP, Fenton C, Bambra C, Bond L. Welfare-to-work interventions and their effects on the mental and physical health of lone parents and their children. Cochrane Database Syst Rev. 2018;2(2):Cd009820. doi: 10.1002/14651858.CD009820.pub3.
18. Goudet SM, Bogin BA, Madise NJ, Griffiths PL. Nutritional interventions for preventing stunting in children (birth to 59 months) living in urban slums in low- and middle-income countries (LMIC). Cochrane Database Syst Rev. 2019;6(6):Cd011695. doi: 10.1002/14651858.CD011695.pub2.
19. Hayes SL, Mann MK, Morgan FM, Kelly MJ, Weightman AL. Collaboration between local health and local government agencies for health improvement. Cochrane Database Syst Rev. 2012;10:Cd007825. doi: 10.1002/14651858.CD007825.pub6.
20. Hollands GJ, Carter P, Anwer S, King SE, Jebb SA, Ogilvie D, Shemilt I, Higgins JPT, Marteau TM. Altering the availability or proximity of food, alcohol, and tobacco products to change their selection and consumption. The Cochrane database of systematic reviews. 2019;9(9):CD012573-CD012573. doi: 10.1002/14651858.CD012573.pub3.
21. Hollands GJ, Shemilt I, Marteau TM, Jebb SA, Lewis HB, Wei Y, Higgins JP, Ogilvie D. Portion, package or tableware size for changing selection and consumption of food, alcohol and tobacco. Cochrane Database Syst Rev. 2015;2015(9):Cd011045. doi: 10.1002/14651858.CD011045.pub2.
22. Hombali AS, Solon JA, Venkatesh BT, Nair NS, Peña-Rosas JP. Fortification of staple foods with vitamin A for vitamin A deficiency. Cochrane Database Syst Rev. 2019;5(5):Cd010068. doi: 10.1002/14651858.CD010068.pub2.

23. Husk K, Lovell R, Cooper C, Stahl-Timmins W, Garside R. Participation in environmental enhancement and conservation activities for health and well-being in adults: a review of quantitative and qualitative evidence. Cochrane Database Syst Rev. 2016;2016(5):Cd010351. doi: 10.1002/14651858.CD010351.pub2.
24. Joyce K, Pabayo R, Critchley JA, Bambra C. Flexible working conditions and their effects on employee health and wellbeing. Cochrane Database Syst Rev. 2010;2010(2):Cd008009. doi: 10.1002/14651858.CD008009.pub2.
25. Lhachimi SK, Pega F, Heise TL, Fenton C, Gartlehner G, Griebler U, Sommer I, Bombana M, Katikireddi SV. Taxation of the fat content of foods for reducing their consumption and preventing obesity or other adverse health outcomes. Cochrane Database Syst Rev. 2020;9:Cd012415. doi: 10.1002/14651858.CD012415.pub2.
26. MacArthur G, Caldwell DM, Redmore J, Watkins SH, Kipping R, White J, Chittleborough C, Langford R, Er V, Lingam R *et al*. Individual-, family-, and school-level interventions targeting multiple risk behaviours in young people. Cochrane Database Syst Rev. 2018;10(10):Cd009927. doi: 10.1002/14651858.CD009927.pub2.
27. Marx R, Tanner-Smith EE, Davison CM, Ufholz L-A, Freeman J, Shankar R, Newton L, Brown RS, Parpia AS, Cozma I *et al*. Later school start times for supporting the education, health, and well-being of high school students. The Cochrane database of systematic reviews. 2017;7(7):CD009467-CD009467. doi: 10.1002/14651858.CD009467.pub2.
28. McLaren L, Sumar N, Barberio AM, Trieu K, Lorenzetti DL, Tarasuk V, Webster J, Campbell NR. Population-level interventions in government jurisdictions for dietary sodium reduction. Cochrane Database Syst Rev. 2016;9(9):Cd010166. doi: 10.1002/14651858.CD010166.pub2.
29. Mosdøl A, Lidal IB, Straumann GH, Vist GE. Targeted mass media interventions promoting healthy behaviours to reduce risk of non-communicable diseases in adult, ethnic minorities. Cochrane Database Syst Rev. 2017;2(2):Cd011683. doi: 10.1002/14651858.CD011683.pub2.
30. Murtagh EM, Murphy MH, Milton K, Roberts NW, O'Gorman CS, Foster C. Interventions outside the workplace for reducing sedentary behaviour in adults under 60 years of age. Cochrane Database Syst Rev. 2020;7(7):Cd012554. doi: 10.1002/14651858.CD012554.pub2.
31. Naude CE, Brand A, Schoonees A, Nguyen KA, Chaplin M, Volmink J. Low-carbohydrate versus balanced-carbohydrate diets for reducing weight and cardiovascular risk. Cochrane Database Syst Rev. 2022;1(1):Cd013334. doi: 10.1002/14651858.CD013334.pub2.
32. Pega F, Liu SY, Walter S, Lhachimi SK. Unconditional cash transfers for assistance in humanitarian disasters: effect on use of health services and health outcomes in low- and middle-income countries. Cochrane Database Syst Rev. 2015;(9):Cd011247. doi: 10.1002/14651858.CD011247.pub2.
33. Pega F, Liu SY, Walter S, Pabayo R, Saith R, Lhachimi SK. Unconditional cash transfers for reducing poverty and vulnerabilities: effect on use of health services and health outcomes in low- and middle-income countries. Cochrane Database Syst Rev. 2017;11(11):Cd011135. doi: 10.1002/14651858.CD011135.pub2.
34. Peña-Rosas JP, Mithra P, Unnikrishnan B, Kumar N, De-Regil LM, Nair NS, Garcia-Casal MN, Solon JA. Fortification of rice with vitamins and minerals for addressing

micronutrient malnutrition. Cochrane Database Syst Rev. 2019;2019(10). doi: 10.1002/14651858.CD009902.pub2.
35. Petkovic J, Duench S, Trawin J, Dewidar O, Pardo Pardo J, Simeon R, DesMeules M, Gagnon D, Hatcher Roberts J, Hossain A *et al*. Behavioural interventions delivered through interactive social media for health behaviour change, health outcomes, and health equity in the adult population. Cochrane Database Syst Rev. 2021;5(5):Cd012932. doi: 10.1002/14651858.CD012932.pub2.
36. Pfinder M, Heise TL, Hilton Boon M, Pega F, Fenton C, Griebler U, Gartlehner G, Sommer I, Katikireddi SV, Lhachimi SK. Taxation of unprocessed sugar or sugar-added foods for reducing their consumption and preventing obesity or other adverse health outcomes. Cochrane Database Syst Rev. 2020;4(4):Cd012333. doi: 10.1002/14651858.CD012333.pub2.
37. Santos JAR, Christoforou A, Trieu K, McKenzie BL, Downs S, Billot L, Webster J, Li M. Iodine fortification of foods and condiments, other than salt, for preventing iodine deficiency disorders. Cochrane Database Syst Rev. 2019;2(2):Cd010734. doi: 10.1002/14651858.CD010734.pub2.
38. Shah D, Sachdev HS, Gera T, De-Regil LM, Peña-Rosas JP. Fortification of staple foods with zinc for improving zinc status and other health outcomes in the general population. Cochrane Database Syst Rev. 2016;2016(6):Cd010697. doi: 10.1002/14651858.CD010697.pub2.
39. Thomson H, Thomas S, Sellstrom E, Petticrew M. Housing improvements for health and associated socio-economic outcomes. Cochrane Database Syst Rev. 2013;(2):Cd008657. doi: 10.1002/14651858.CD008657.pub2.
40. Virgara R, Phillips A, Lewis LK, Baldock K, Wolfenden L, Ferguson T, Richardson M, Okely A, Beets M, Maher C. Interventions in outside-school hours childcare settings for promoting physical activity amongst schoolchildren aged 4 to 12 years. Cochrane Database Syst Rev. 2021;9(9):Cd013380. doi: 10.1002/14651858.CD013380.pub2.
41. Visser ME, Schoonees A, Ezekiel CN, Randall NP, Naude CE. Agricultural and nutritional education interventions for reducing aflatoxin exposure to improve infant and child growth in low- and middle-income countries. Cochrane Database Syst Rev. 2020;4(4):Cd013376. doi: 10.1002/14651858.CD013376.pub2.
42. von Philipsborn P, Stratil JM, Burns J, Busert LK, Pfadenhauer LM, Polus S, Holzapfel C, Hauner H, Rehfuess E. Environmental interventions to reduce the consumption of sugar-sweetened beverages and their effects on health. Cochrane Database Syst Rev. 2019;6(6):Cd012292. doi: 10.1002/14651858.CD012292.pub2.
43. Wolfenden L, Barnes C, Jones J, Finch M, Wyse RJ, Kingsland M, Tzelepis F, Grady A, Hodder RK, Booth D *et al*. Strategies to improve the implementation of healthy eating, physical activity and obesity prevention policies, practices or programmes within childcare services. Cochrane Database Syst Rev. 2020;2(2):Cd011779. doi: 10.1002/14651858.CD011779.pub3.
44. Wolfenden L, Goldman S, Stacey FG, Grady A, Kingsland M, Williams CM, Wiggers J, Milat A, Rissel C, Bauman A *et al*. Strategies to improve the implementation of workplace-based policies or practices targeting tobacco, alcohol, diet, physical activity and obesity. Cochrane Database Syst Rev. 2018;11(11):Cd012439. doi: 10.1002/14651858.CD012439.pub2.
45. Wolfenden L, Nathan NK, Sutherland R, Yoong SL, Hodder RK, Wyse RJ, Delaney T, Grady A, Fielding A, Tzelepis F *et al*. Strategies for enhancing the implementation of

school-based policies or practices targeting risk factors for chronic disease. Cochrane Database Syst Rev. 2017;11(11):Cd011677. doi: 10.1002/14651858.CD011677.pub2.
46. Anglemyer A, Moore TH, Parker L, Chambers T, Grady A, Chiu K, Parry M, Wilczynska M, Flemyng E, Bero L. Digital contact tracing technologies in epidemics: a rapid review. Cochrane Database Syst Rev. 2020;8(8):Cd013699. doi: 10.1002/14651858.Cd013699.
47. Burns J, Movsisyan A, Stratil JM, Biallas RL, Coenen M, Emmert-Fees KM, Geffert K, Hoffmann S, Horstick O, Laxy M *et al*. International travel-related control measures to contain the COVID-19 pandemic: a rapid review. Cochrane Database Syst Rev. 2021;3(3):Cd013717. doi: 10.1002/14651858.CD013717.pub2.
48. Krishnaratne S, Littlecott H, Sell K, Burns J, Rabe JE, Stratil JM, Litwin T, Kreutz C, Coenen M, Geffert K *et al*. Measures implemented in the school setting to contain the COVID-19 pandemic. Cochrane Database Syst Rev. 2022;1(1):Cd015029. doi: 10.1002/14651858.Cd015029.
49. Noone C, McSharry J, Smalle M, Burns A, Dwan K, Devane D, Morrissey EC. Video calls for reducing social isolation and loneliness in older people: a rapid review. Cochrane Database Syst Rev. 2020;5(5):Cd013632. doi: 10.1002/14651858.Cd013632.
50. Stratil JM, Biallas RL, Burns J, Arnold L, Geffert K, Kunzler AM, Monsef I, Stadelmaier J, Wabnitz K, Litwin T *et al*. Non-pharmacological measures implemented in the setting of long-term care facilities to prevent SARS-CoV-2 infections and their consequences: a rapid review. Cochrane Database Syst Rev. 2021;9(9):Cd015085. doi: 10.1002/14651858.CD015085.pub2.
51. Turley R, Saith R, Bhan N, Rehfuess E, Carter B. Slum upgrading strategies involving physical environment and infrastructure interventions and their effects on health and socio-economic outcomes. Cochrane Database Syst Rev. 2013;(1):Cd010067. doi: 10.1002/14651858.CD010067.pub2.
52. Viswanathan M, Kahwati L, Jahn B, Giger K, Dobrescu AI, Hill C, Klerings I, Meixner J, Persad E, Teufer B *et al*. Universal screening for SARS-CoV-2 infection: a rapid review. Cochrane Database Syst Rev. 2020;9(9):Cd013718. doi: 10.1002/14651858.Cd013718.
53. Krishnaratne S, Pfadenhauer LM, Coenen M, Geffert K, Jung-Sievers C, Klinger C, Kratzer S, Littlecott H, Movsisyan A, Rabe JE *et al*. Measures implemented in the school setting to contain the COVID-19 pandemic: a scoping review. Cochrane Database Syst Rev. 2020;12:Cd013812. doi: 10.1002/14651858.Cd013812.
54. Amberson T, Heagele T, Castner J, Wyte-Lake T, Couig MP, Bell SA, Mammen MJ, Wells V. Social support, educational, and behavioral modification interventions for improving household disaster preparedness in the general community-dwelling population. Cochrane Database of Systematic Reviews. 2021;(5). doi: 10.1002/14651858.CD014934.
55. Armstrong R, Waters E, Dobbins M, Lavis JN, Petticrew M, Christensen R. Knowledge translation strategies for facilitating evidence-informed public health decision making among managers and policy-makers. Cochrane Database of Systematic Reviews. 2011;(6). doi: 10.1002/14651858.CD009181.
56. Baker PRA, Dobbins M, Soares J, Francis DP, Weightman AL, Costello JT. Public health interventions for increasing physical activity in children, adolescents and adults: an overview of systematic reviews. Cochrane Database of Systematic Reviews. 2015;(1). doi: 10.1002/14651858.CD011454.
57. Burns C, Kristjansson B, Harris G, Armstrong R, Cummins S, Black A, Lawrence M. Community level interventions to improve food security in developed countries.

Cochrane Database of Systematic Reviews. 2010;(12). doi: 10.1002/14651858.CD008913.
58. Clarke N, Marteau TM, Pilling M, Roberts NW, Jebb SA, Hollands GJ. Energy (calorie) labelling for healthier selection and consumption of food or alcohol. Cochrane Database of Systematic Reviews. 2021;(6). doi: 10.1002/14651858.CD014845.
59. Cukier S, Wettlaufer A, Jackson K, Minozzi S, Bartholow BD, Stoolmiller ML, Sargent JD. Impact of exposure to alcohol marketing and subsequent drinking patterns among youth and young adults. Cochrane Database of Systematic Reviews. 2018;(8). doi: 10.1002/14651858.CD013087.
60. Flatz A, Pfeifer N, Radtke T, Kriemler S, Klerings I, Wolfenden L, von Elm E. Interventions implemented through sporting organisations for promoting healthy behaviour or improving health outcomes. Cochrane Database of Systematic Reviews. 2016;(5). doi: 10.1002/14651858.CD012170.
61. Garcia-Casal MN, Peña-Rosas JP, Pachón H, De-Regil LM, Centeno Tablante E, Flores-Urrutia MC. Staple crops biofortified with increased micronutrient content: effects on vitamin and mineral status, as well as health and cognitive function in the general population. Cochrane Database of Systematic Reviews. 2016;(8). doi: 10.1002/14651858.CD012311.
62. Harris R, Raison H, Christian B, Bakare L, Okwundu CI, Burnside G. Interventions for improving adults' use of primary oral health care services. Cochrane Database of Systematic Reviews. 2017;(8). doi: 10.1002/14651858.CD012771.
63. Heise TL, Katikireddi SV, Pega F, Gartlehner G, Fenton C, Griebler U, Sommer I, Pfinder M, Lhachimi SK. Taxation of sugar-sweetened beverages for reducing their consumption and preventing obesity or other adverse health outcomes. Cochrane Database of Systematic Reviews. 2016;(8). doi: 10.1002/14651858.CD012319.
64. O'Malley GC, Baker PRA, Francis DP, Perry I, Foster C. Incentive-based interventions for increasing physical activity and fitness. Cochrane Database of Systematic Reviews. 2012;(1). doi: 10.1002/14651858.CD009598.
65. Piper JD, Chandna J, Allen E, Linkman K, Cumming O, Prendergast AJ, Gladstone MJ. Water, sanitation and hygiene (WASH) interventions: effects on child development in low- and middle-income countries. Cochrane Database of Systematic Reviews. 2017;(3). doi: 10.1002/14651858.CD012613.
66. Self JL, Serdula M, Dowswell T, De-Regil LM. Fortification of condiments and seasonings with iron for preventing anaemia and improving health. Cochrane Database of Systematic Reviews. 2012;(2). doi: 10.1002/14651858.CD009604.
67. Tollit MA, Sawyer SM, Ratnapalan S, Barnett T. Education support services for improving school engagement and academic performance of children and adolescents with a chronic health condition. Cochrane Database of Systematic Reviews. 2015;(2). doi: 10.1002/14651858.CD011538.
68. Yoong SL, Lum M, Jackson J, Wolfenden L, Barnes C, Jones J, Pearson N, McCrabb S, Hall AE, Leonard A *et al*. Healthy eating interventions delivered in early childhood education and care settings for improving the diet of children aged six years and below. Cochrane Database of Systematic Reviews. 2021;(2). doi: 10.1002/14651858.CD013862.

Table S4. Plain language summary

Language	Summary
English	This study aimed to find out how the results of Cochrane Public Health reviews are communicated. We searched all 68 reviews from the Cochrane Public Health website. Article authors rarely explained how they will disseminate their results. All publications included plain languages summaries. All plain language summaries were available in English, German and Spanish. Most reviews also used other dissemination strategies available on Cochrane websites such as clincal answers or podcasts. Reviews should be widely communicated. They are interesting for the general population and non-academic professionals.
German	In dieser Studie wurde untersucht, wie die Ergebnisse von Cochrane-Reviews zur Gesundheit der Bevölkerung verbreitet werden. Wir untersuchten 68 Cochrane-Reviews. Die Autor*innen der Reviews erklärten nur selten, wie sie ihre Ergebnisse verbreiten werden. Alle Reviews haben aber Zusammenfassungen in einfacher Sprache in Englisch, Deutsch, Spanisch und zum Teil in anderen Sprachen. Zu vielen Reviews gibt es auch weitere Texte und Kommunikationsformate auf der Cochrane-Website. Die untersuchten Reviews sind für die allgemeine Bevölkerung und nichtakademische Fachleute in verschiedenen Bereichen wie Ernährung, Sport, Bildung, Politik und Pflege interessant. Wie man die betroffenen Personen möglichst gut erreicht, muss weiter untersucht werden.
